# Hemorrhage

**Published:** 1861-10

**Authors:** 


### HEMORRHAGE.

Dr. H. Collins says, that for the treatment of hemorrhage after the extraction of teeth, he uses Plaster Paris, mixed as usual, with some fibres of cotton or flax worked into it; then when it is of the proper consistence plug up the socket with it, and the hemorrhage will at once be arrested. He says no styptic is required with it. T.

